# Stochastic approach to study control strategies of Covid-19 pandemic in India

**DOI:** 10.1017/S0950268820001946

**Published:** 2020-08-28

**Authors:** Athokpam Langlen Chanu, R. K. Brojen Singh

**Affiliations:** School of Computational & Integrative Sciences, Jawaharlal Nehru University, New Delhi 110067, India

**Keywords:** Covid-19 in India, quarantine, social distancing, stochastic modelling, stochastic simulation algorithm

## Abstract

India is one of the severely affected countries by the Covid-19 pandemic at present. Within the stochastic framework of the SEQIR model, we studied publicly available data of the Covid-19 patients in India and analysed possible impacts of quarantine and social distancing as controlling strategies for the pandemic. Our stochastic simulation results clearly show that proper quarantine and social distancing should be maintained right from the start of the pandemic and continued until its end for effective control. This calls for a more disciplined social lifestyle in the future. However, only social distancing and quarantine of the exposed population are found not sufficient enough to end the pandemic in India. Therefore, implementation of other stringent policies like complete lockdown as well as increased testing of susceptible populations is necessary. The demographic stochasticity, which is quite visible in the system dynamics, has a critical role in regulating and controlling the pandemic.

## Introduction

The novel coronavirus disease 2019 (Covid-19) is a highly infectious disease caused by the SARS-CoV-2 virus, which can lead to severe complications such as Covid-19 pneumonia [1]. On 11 March 2020, the World Health Organization (WHO) declared the Covid-19 outbreak as a pandemic of special health attention [2]. As of 2 June 2020 (10:06 am CEST), the total number of confirmed cases globally is 6 140 934 with 373 548 deaths [2]. India is now one of the countries severely affected by the Covid-19 pandemic. As of 2 June 2020 (7:45 pm IST), the total number of Covid-19 confirmed cases in India is 201 341 with 99 135 active cases and 5632 deaths [3]. The present situation of the pandemic is alarming since there is no vaccine/drug developed to cure this disease. So far, the Indian Government, both at the central and state levels, has taken up special measures such as quarantine, social distancing and lockdown to prevent/intervene in the spread of Covid-19 across the country.

Mathematical modelling of epidemics plays important roles to study and predict disease dynamics as well as to suggest necessary intervention strategies for controlling disease outbreaks. Classic compartmental models such as SI, SIS, SIR [4], SEIR and their derived/extended models [5] have long been successfully used to study various disease transmission dynamics for different viruses such as the H1N1 virus [6], the Ebola virus [7], SARS-CoV [8], MERS-CoV [9], etc. With special reference to the ongoing Covid-19 pandemic, there have been many attempts using statistical methods, deterministic compartmental modelling, large-scale simulation to study Covid-19 disease dynamics and to propose policies to intervene in the disease outbreak in several countries. Likewise, there have been many studies on the Covid-19 pandemic in India using various mathematical models. However, most of these studies adopt deterministic methods [10–21]. On the other hand, to capture the qualitative as well as quantitative real dynamic situations to intervene in disease outbreaks, a stochastic approach needs to be employed. With the increasing capacity of modern computers, stochastic methods are gaining popularity because they are powerful methods to study and predict any dynamic complex system that has inherently various environmental fluctuations/noise. A stochastic approach towards disease transmission models becomes especially important in situations such as the beginning or the end of a disease outbreak when there are less infective individuals. In such situations, stochasticity is non-negligible and may play an important role in system dynamics. The objective of this paper is to study the Covid-19 disease dynamics in India and some of its states using the SEQIR model from a stochastic approach. Using stochastic numerical simulations, we illustrate important impacts of social distancing and quarantine on the Covid-19 transmission in India and its five states, namely Uttar Pradesh, Delhi, Kerala, Maharashtra and West Bengal. We also highlight the importance of demographic stochasticity in Covid-19 spreading dynamics.

## Methods

### SEQIR model

**Fig. 1.**
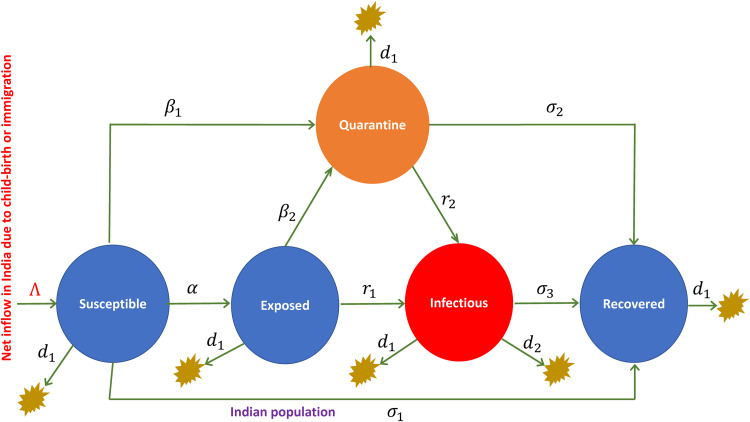
The schematic diagram of the SEQIR model (adapted from the reference [17]).

Most of the states in India adopt home quarantine and institutional quarantine policies to reduce the transmission of Covid-19. The classic SEIR (Susceptible, Exposed, Infected and Recovered) epidemic model [5] is therefore extended with another compartment called Quarantine (Q) to study the Covid-19 disease dynamics in India. The extended model is known as the SEQIR model [17], its schematic diagram shown in Figure 1. In this model (Fig. 1), the total population is sub-divided into five sub-populations (compartments) such as Susceptible (S); Exposed and infected but undetected by testing (E); Quarantined (Q); confirmed/reported/hospitalised Infected population (I); and Covid-19 recovered patients as well as individuals living in secured zones unaffected by the Covid-19 outbreak (R). In Figure 1, *α* is the rate of Covid-19 transmission per individual; *β*_1_ is the rate of transition from Susceptible to the Quarantined class; *σ*_1_ is the transition rate from Susceptible to the secured zone (unaffected by Covid-19) or recovered class; *β*_2_ is the quarantine rate of the Exposed individuals who are infected but undetected by testing; *r*_1_ represents the rate of progression from the Exposed and infected but undetected by testing class to the hospitalised Infected class; *σ*_2_ is the rate of transition from Quarantined to the secured zone class, or the natural recovery rate of some infected Quarantined individuals; *r*_2_ is the transition rate of some infected Quarantined individuals to hospitalised Infected class; *σ*_3_ denotes the recovery rate of the hospitalised Infected class; *d*_2_ indicates the Covid-19 induced death rate; Λ is the rate of inflow in the Indian population due to new child-births or immigration to the country; and *d*_1_ denotes the natural death rate of each class. We assume uniform mixing or homogeneity in the large population. Consider the total population at any instant of time is *N*(*t*) = *S*(*t*) + *E*(*t*) + *Q*(*t*) + *I*(*t*) + *R*(*t*).

### Stochastic modelling

Change in population occurs in discrete integer amounts and is a stochastic process. Hence, the time evolution of each variable (sub-population) in the model should be considered in a discrete and stochastic fashion rather than in a deterministic manner [22]. In the stochastic formalism of the SEQIR model of Figure 1, the population state vector at any instant of time can be represented by, ***X*** = [*S*, *E*, *Q*, *I*, *R*]^−1^. We assume the set of sub-populations {*X_i_*} = {*S*, *E*, *Q*, *I*, *R*} to be a set of molecular species. For the SEQIR model of Figure 1, the state vector ***X*** undergoes *M* = 15 reaction channels defined by, 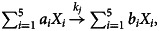 where *k_j_* represents the classical rate constants. Here, sets {*a_i_*} and {*b_i_*} are the sets of reactant and product molecules, respectively. Further, the classical rate constant, *k_j_* relates to the stochastic rate constant, *c_j_* as per relation, *c*_*j*_ = *k*_*j*_*V*^1−*ν*^, where *ν* denotes the stoichiometric ratio and *V* is the system size [22, 23]. This relation incorporates the idea of correlating fluctuations in the dynamics of the system [22–24]. Now, the reaction channels translated from Figure 1 are as follows,1
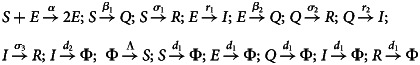


If the system is subjected to a certain temperature *T*, then any variable in ***X*** undergoes a set of random molecular events, given by the set of reactions (1). Thus, the trajectory of the variable in ***X*** follows the well-known Brownian motion [22–24]. Further, each time anyone of the reactions in the reaction channels (1) is encountered, the creation and annihilation of the molecular species will occur and hence, the state vector ***X*** will change as a function of time. Now, consider the state vector ***X*** changes to another state ***X*^’^** during the time interval [*t*, *t* + Δ*t*]. Then, the time evolution of the configurational probability of state change *P* (***X***; *t*) is given by the following Master equation, constructed from the detailed-balance equation [25–27],2
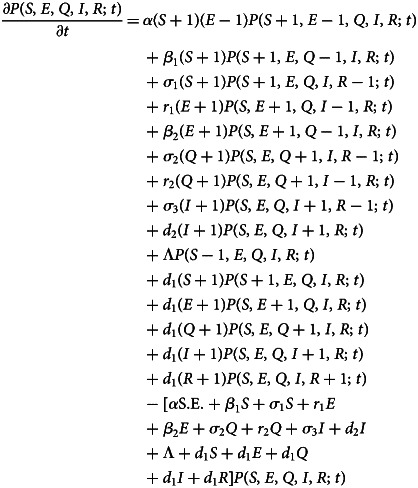


Except for simple systems like linear systems, it is usually difficult to analytically solve a multivariate Master equation such as equation (2). However, the multivariate Master equation (2) can be solved numerically using the Stochastic Simulation Algorithm (SSA), briefly discussed here. The SSA is generally known as the Doob–Gillespie algorithm. It is formulated by Gillespie [22, 24] on the basis of the theoretical foundations given by Doob [28, 29] and originally proposed by Kendall [30]. It is a Monte-Carlo type of algorithm, which is a non-spatial individual-based analogue of the Master equation that incorporates all possible interactions in the system [22]. The SSA is built on two random processes which are statistically independent, namely firing reaction and reaction time. In SSA, these two processes are realised by generating two statistically independent, uniform random numbers *r*_1_ and *r*_2_. The reaction time *τ* is computed using *τ* = (1/*a*_0_)ln(1/*r*_1_). Here, *a*_0_ is given by, 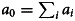, where *a_i_* represents the *i*^th^ propensity function. The propensity function, *a_i_* relates to the stochastic rate constant, *c*_*i*_according to the formula *a*_*i*_ = *h*_*i*_*c*_*i*_, where *h_i_* denotes the number of possible molecular combinations of *i*^th^ reaction. Further, the *j*^th^ reaction will fire, when it satisfies, 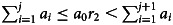.

### *R*_0_ calculation using a deterministic approach

For compartmental epidemiological models with a set of Ordinary Differential Equations (ODEs), the basic reproduction number *R*_0_ is usually determined by the *Next Generation Matrix* method, proposed by Diekmann *et al*. [31]. Here, we will only give a rough calculation of *R*_0_ without going into the details of mathematical proofs and analysis. One can see the references [32, 33] for the detailed proofs and analysis of *R*_0_ calculation. For the SEQIR model in Figure 1, the *R*_0_ can be determined as follows. Let *ω* = [*E*, *Q*, *I*, *R*, *S*]^*T*^be the population state vector representing the population in each compartment of Figure 1. Consider the first *m* < *n* compartments have individuals infected by the SARS-CoV-2 virus, where *m* = 3 (i.e., *E*, *Q*, *I*) and *n* = 5 (i.e., *E*, *Q*, *I*, *R*, *S*) for the model system. Assume that the Disease Free Equilibrium (DFE) *ω*_0_ exists for the model. Then, *ω*_0_ = (0, 0, 0, 0, *S*_0_), where *S*_0_ = (Λ/*β*_1_ + *σ*_1_ + *d*_1_), which is a fraction of Susceptible individuals when Covid-19 is not present. We can rewrite the governing ODEs of the model in Figure 1 as (d*ω*_*i*_/d*t*) = Γ_*i*_(*ω*) − Θ _*i*_(*ω*), where *i* = 1, 2, 3 (or *E*, *Q*, *I*). Here, the function Γ_*i*_(*ω*) describes the rate of new infections appearing in the compartment *i*, and the function Θ _*i*_(*ω*) is the rate of all possible transitions between the compartment *i* and any other infected compartments [32, 33]. So, for the SEQIR model in Figure 1, we calculated these two functions as, 
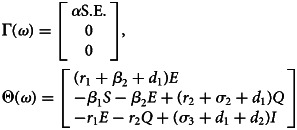
With the definitions 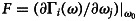 and 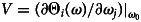 for 1 ⩽ *i*, *j* ⩽ *m*, the *Next Generation Matrix* is given by the product *FV*^−1^. The spectral radius of the *Next Generation Matrix* then gives the basic reproduction number *R*_0_, i.e. *R*_0_ = ϱ(*FV*^−1^), where ϱ represents the spectral radius [32]. The *Next Generation Matrix FV*^−1^ has the (*i*, *j*) entry equal to the expected number of secondary infections in compartment *i* produced by an infected individual introduced in compartment *j* [33]. For the model system in Figure 1, we calculated,
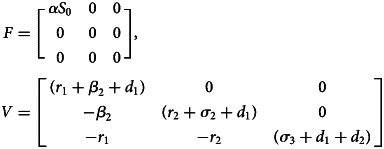


Then, the eigenvalues of the *Next Generation Matrix FV*^−1^ are 0, 0 and *α*Λ/(*β*_1_ + *σ*_1_ + *d*_1_)(*r*_1_ + *β*_2_ + *d*_1_). Thus, the basic reproduction number is calculated to be *R*_0_ = (*α*Λ/(*β*_1_ + *σ*_1_ + *d*_1_)(*r*_1_ + *β*_2_ + *d*_1_)) (similarly reported in [17]). Rewriting, *R*_0_ = *S*_0_ × *α* × (1/*r*_1_ + *β*_2_ + *d*_1_) = Susceptible population at DFE × transmissibility × net duration of infectiousness, quarantining and natural death rate.

## Results and discussion

With a deterministic approach, we calculated the basic reproduction number *R*_0_ = (Λ*α*/(*β*_1_ + *σ*_1_ + *d*_1_ )(*r*_1_ + *β*_2_ + *d*_1_ )). We then made rough estimates of *R*_0_ values for India and its five states, namely Uttar Pradesh, Delhi, Kerala, Maharashtra and West Bengal using the values of transition rates given in Table 1 (the primary sources of data are [2, 34, 35]). As of 21 March 2020, the *R*_0_ values of India, Uttar Pradesh, Delhi, Kerala, Maharashtra and West Bengal are, respectively, 0.1747, 21.81, 0.4862, 0.2329, 0.3556 and 0.5774. This mentioned date particularly falls during an early stage of the Covid-19 outbreak in all the five states and also for India, when there are initially less infected individuals (see Table 2). The infected population is less than 100 in all the five states and is less than 300 for India. So, we imposed a stochastic approach to the SEQIR model of Figure 1, which takes into account the stochasticity that is present in the system dynamics.

**Table 1.** Rate constant values taken from [17]

Rate constants (per day)	India	Maharashtra	Kerala	Delhi	Uttar Pradesh	West Bengal
α	0.00000000025	0.0000000025	0.0000000164	0.000000021	0.00000002	0.0000000047
*β*_1_	0.0000004	0.0000004	0.0000004	0.0000004	0.0000004	0.0000004
*σ*_1_	0.0005	0.0005	0.0005	0.0005	0.0005	0.0005
*r*_1_	0.01	0.04	0.05	0.05	0.02	0.05
*β*_2_	0.1	0.005	0.005	0.005	0.005	0.005
*σ*_2_	0.05	0.1	0.1	0.1	0.1	0.1
*r*_2_	0.001	0.002	0.001	0.00093	0.0012	0.0005
*σ*_3_	0.006	0.005	0.012	0.0016	0.0038	0.0078
*d*_2_	0.00197	0.0032	0.00029	0.00067	0.0049	0.0024
Λ	40 000	3300	405	650	14 200	3490
*d*_1_	0.00002	0.000015	0.000018	0.00001	0.00002	0.000016

**Table 2.** Initial values taken from [17]

Initial values	India	Maharashtra	Kerala	Delhi	Uttar Pradesh	West Bengal
*S*(0)	800 000 000	75 000 000	10 000 000	10 000 000	150 000 000	50 000 000
*E*(0)	1500	225	200	150	120	20
*Q*(0)	50 000	800	1000	800	1500	200
*I*(0)	284	58	40	27	24	3
*R*(0)	400 000 000	30 000 000	10 050 000	500 000	50 000 000	30 000 000

**Fig. 2.**
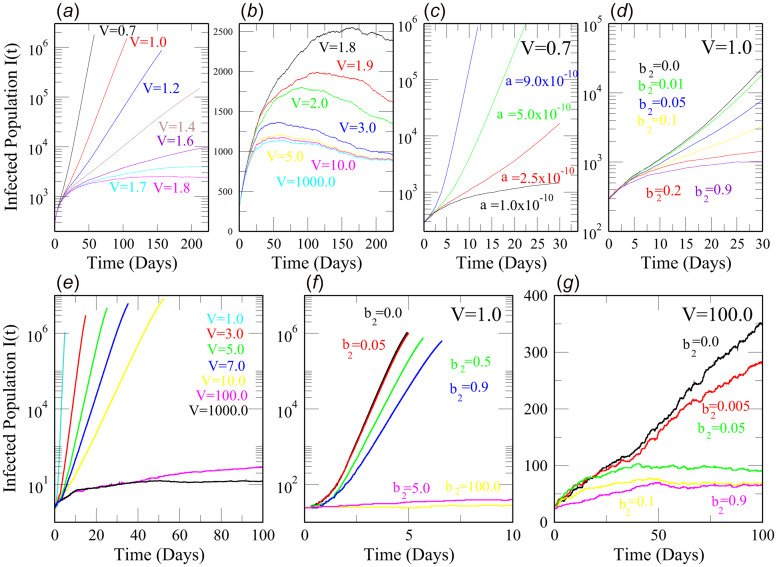
The upper panels (a–d) represent the simulation results of Infected Population *I*(*t*) *vs.* time, *t* (in days) for India using the Stochastic Simulation Algorithm (SSA). (a and b) *I*(*t*) *vs. t* for India for different values of the system size *V*. As *V* increases, *I*(*t*) decreases. (c) *I*(*t*) *vs. t* for India for different values of transmission rate *α* at *V* = 0.7. *I*(*t*) increases with increase in *α*. (d) *I*(*t*) *vs. t* for India for different values of quarantine rate *β*_2_ at *V* = 1.0. *I*(*t*) decreases with increase in *β*_2_. The lower panels (e–g) represent the simulation results of Infected Population *I*(*t*) *vs.* time *t* (in days) for Uttar Pradesh using the SSA. (e) *I*(*t*) *vs. t* for Uttar Pradesh for different values of *V*. As *V* increases, *I*(*t*) decreases. (f and g) *I*(*t*) *vs. t* for Uttar Pradesh for different values of quarantine rates *β*_2_ at two values of system size *V* = 1.0 and *V* = 100.0, respectively. In both (f) and (g), *I*(*t*) decreases with increase in *β*_2_.

**Fig. 3.**
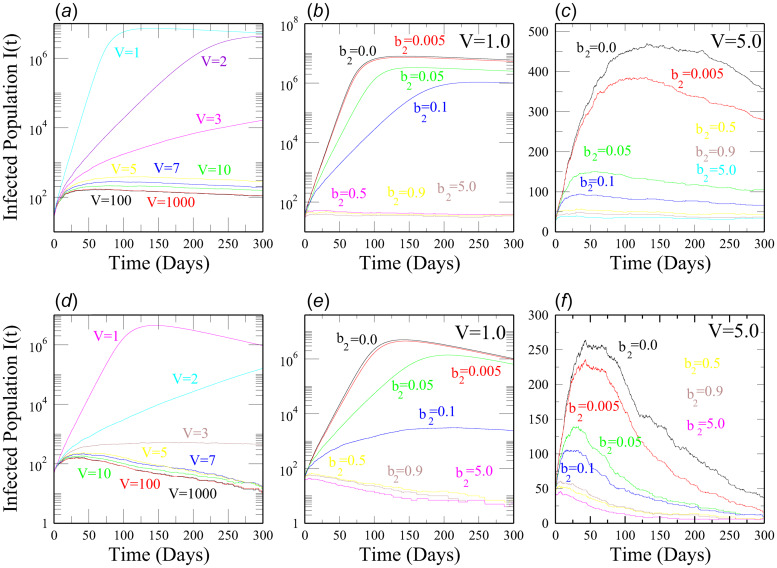
The upper panels (a–c) show simulation results of Infected Population *I*(*t*) *vs.* time *t* (in days) for Delhi using the Stochastic Simulation Algorithm (SSA). (a) *I*(*t*) *vs. t* of Delhi for different values of the system size *V*. As *V* increases, *I*(*t*) decreases. (b and c) *I*(*t*) *vs. t* of Delhi for different values of quarantine rate *β*_2_ at two different volumes *V* = 1.0 and *V* = 5.0, respectively. In both (b) and (c), *I*(*t*) decreases with increase in *β*_2_. Again, (d–f) show simulation results of Infected Population *I*(*t*) *vs.* time *t* (in days) for Kerala using SSA. (d) *I*(*t*) *vs. t* of Kerala for different values of *V*. As *V* increases, *I*(*t*) decreases. (e and f) *I*(*t*) *vs. t* of Kerala for different values of quarantine rate *β*_2_ at two different volumes *V* = 1.0 and *V* = 5.0, respectively. In both (e) and (f), *I*(*t*) decreases with increase in *β*_2_.

**Fig. 4.**
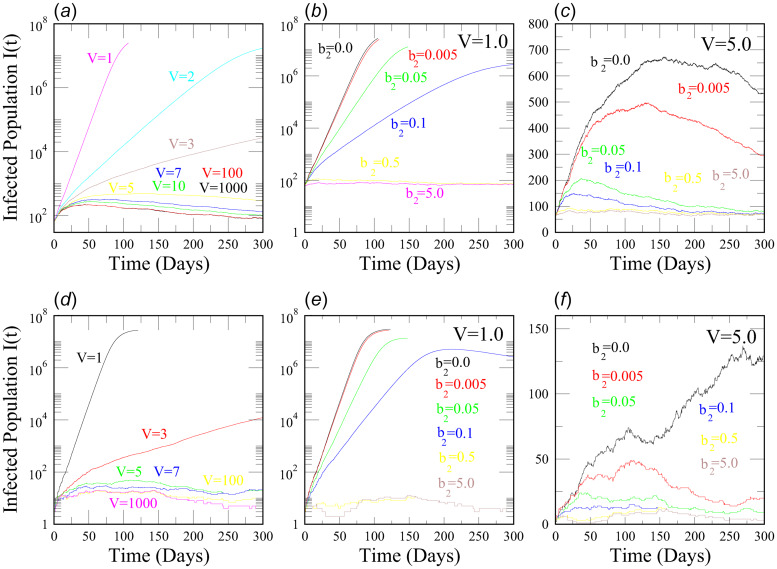
The upper panels (a–c) show simulation results of Infected Population *I*(*t*) *vs.* time *t* (in days) for Maharashtra using Stochastic Simulation Algorithm. (a) *I*(*t*) *vs. t* of Maharashtra for different values of system size *V*. *I*(*t*) decreases with increase in *V*. (b and c) *I*(*t*) *vs. t* of Maharashtra for different values of quarantine rate *β*_2_ at two different volumes *V* = 1.0 and *V* = 5.0, respectively. In both (b) and (c), *I*(*t*) decreases with increase in *β*_2_. Again, (d–f) show simulation results of Infected Population *I*(*t*) *vs.* time *t* (in days) for West Bengal using the SSA. (d) *I*(*t*) *vs. t* of West Bengal for different values of *V*. *I*(*t*) decreases with increase in *V*. (e and f) *I*(*t*) *vs. t* of West Bengal for different values of *β*_2_ at two different volumes *V* = 1.0 and *V* = 5.0, respectively. In both (e) and (f), *I*(*t*) decreases with increase in *β*_2_.

We performed numerical simulations of the SEQIR model in Figure 1 using the SSA to study the time evolution of the Covid-19-infected population *I*(*t*) for India and the above mentioned five Indian states with respect to parameters such as transmission rate (*α*), quarantine rate (*β*_2_) and system size (*V*). We considered the mentioned five Indian states because we wanted to study the Covid-19 disease dynamics in these densely populated states as compared to other Indian states. In population dynamics, one can express the system size parameter *V* as, *V* = *N*/(*N*/*V*) = *N*/*D*; where *N* is the total population in the geographical area, and *D* is the population density. Taking *N* as constant, we can correlate the change in *V* as a change in *D* by the relation, *V* ∝ (1/D). The rate constant values and initial values for the SSA simulation are taken from the reference [17] (see Tables 1 and 2) after verifying the data from 21 March 2020 to 2 June 2020 [3]. The SSA simulation results of *I*(*t*) *vs.* time, *t* (in days) for India, Uttar Pradesh, Delhi, Kerala, Maharashtra and West Bengal are shown in Figures 2(a–g), 3(a–f), 4(a–f). We analysed the simulation results and provided possible predictions of the Covid-19 pandemic in India and the five states, as presented below.

*Scenario 1 (India):* We numerically simulated the time-evolution of the Covid-19-infected population *I*(*t*) in India using SSA under different conditions. From the simulation results of the dynamics of *I*(*t*) for various values of the system size V, we observed that at values of *V* < 1.7 (or *D* > *N*/1.7), the *I*(*t*) increases exponentially, indicating a monotonic rise in infection (Malthusian law [36] ). The *I*(*t*) curves start flattening (Gompertz-Winsor nature [35]) for *V* ≥ 1.7 (or *D* ≤ *N*/1.7), which is the signature of an endemic (Fig. 2 (b)). Therefore, the infected Indian population *I*(*t*) is greatly influenced by the population density *D* (Fig. 2(a) and (b)), and is not homogeneously distributed over India. Fluctuations arising in the system dynamics due to population density *D* play an important role to intervene in Covid-19 spreading. Hence, these simulation results (Fig. 2(a) and (b)) predict that controlling the population density can lead to an endemic of the Covid-19 outbreak in India. The strategy to decrease population density *D* could be policies such as social distancing, closure of socially active places such as academic institutions, offices, tourist spots, places of worship like temples, churches, mosques, etc.

In Figure 2(c), the sensitivity of the SEQIR model in Figure 1 is studied with regard to the parameter *α*, which is the Covid-19 transmission rate, at a particular system size value *V* = 0.7. As the value of *α* increases, the infected population *I*(*t*) increases sharply. This corresponds to the fact that, as the transmission rate increases due to homogeneous mixing of the population in a certain demographic region, more susceptible *S* population gets exposed and infected with Covid-19. Further, in Figure 2(d), we studied the impact of the quarantine rate, *β*_2_ on the infected population *I*(*t*). One can interpret the quarantine rate, *β*_2_ ~ (1/delay in quarantine). In Figure 2(d), we observed that as the quarantine rate increases (or the delay in quarantine decreases), the *I*(*t*) starts decreasing sharply. When there is no quarantine or *β*_2_ = 0.0, then *I*(*t*) ~ *O*(10^4^). However, when *β*_2_ = 0.2 (quarantine in 5 days), then *I*(*t*) starts flattening around ~*O*(10^3^). This result illustrates the important effects of quarantine on the Covid-19-infected population *I*(*t*) in India. It indicates that quarantining of exposed population *E* needs to be done as quickly as possible and this should be imposed throughout the pandemic to mitigate the Covid-19 spreading in the country. We know that flattening the curve can prevent the burden in hospitals and health care facilities which in turn will keep the pandemic under control. Thus, our SSA simulation results suggest that interventions such as social distancing and quarantine need to be strongly imposed to control the Covid-19 pandemic in a country with a relatively higher population like India.

*Scenario 2 (Uttar Pradesh):* Uttar Pradesh is the fourth largest Indian state. In Figure 2(e), at around *t* = 20 days, *I*(*t*) ~ *O*(10^5^) (when *V* = 5 or *D* = *N*/5); *I*(*t*) ~ *O*(10^4^) (when *V* = 7 or *D* = *N*/7); *I*(*t*) ~ *O*(10^3^) (when *V* = 10 or *D* = *N*/10). This decline in infected population *I*(*t*) with an increase in system size *V* (or correspondingly a decrease in population density *D*) can be attributed to policy such as social distancing, as mentioned before. When *V* ≈ 100 (or *D* ≈ *N*/100), the curve of *I*(*t*) starts flattening, which indicates a well-intervention in the Covid-19 pandemic in the state. Thus, the demographic stochasticity as measured by 1/*V*^1/2^ ∝ *D*^1/2^ can control the time evolution of the infected population *I*(*t*). In Figure 2(f) and (g), we studied the effect of quarantine (*β*_2_) on *I*(*t*) evolution at two fixed values of the system size parameter, i.e. *V* = 1.0 (or *D* = *N*) and *V* = 100 (or *D* = *N*/100). In Figure 2(f), at *V* = 1.0, when there is no quarantine (*β*_2_ = 0.0), the peak of *I*(*t*) ~ *O*(10^6^) and if the quarantine rate *β*_2_ ≥ 5.0, there are roughly 50 infected population in our simulation result. This implies that quick quarantining of the exposed population *E* (in hours) is necessary to control the fast transmission of Covid-19 in a large state with a dense population like Uttar Pradesh. Again, in Figure 2(g), at *V* = 100, when quarantine rate *β*_2_ = 0.0, the peak of *I*(*t*) ~ 350 and if *β*_2_ ≥ 0.1, then our simulation results show approximately 50 infected population. Thus, for Uttar Pradesh, if the exposed population *E* is quarantined in 10 days, then the Covid-19 outbreak is relatively controlled, provided a policy for less population density like social distancing is already strongly imposed. These simulation results thus highlight that the policies such as social distancing and quarantine should be strictly imposed in Uttar Pradesh, otherwise, the Covid-19 outbreak in the state can worsen in a short amount of time, as predicted by Uttar Pradesh's high *R*_0_ value of 21.81.

*Scenario 3 (Delhi):* The SSA simulation results for Delhi show that the peak value of the Covid-19-infected population *I*(*t*) varies drastically with a change in the values of the system size *V*. In Figure 3(a), when *V* = 1 (or *D* = *N*), the peak of *I*(*t*) ~ *O*(10^6^), but when *V* ≈ 5 (or *D* ≈ *N*/5), the peak of *I*(*t*) ~ *O*(10^2^). Flattening of the *I*(*t*) curve with less peak value is achieved if the values of *V* are increased. The number density *D* of the population decreases when *V* is increased since the total population *N* is fixed. Thus, the simulation results imply the significance of decreasing population density. These results clearly suggest that social distancing plays a crucial role in the early times of the pandemic for proper intervention. In Figure 3(b) and (c), the dynamics of *I*(*t*) is studied for different quarantine rates at two system size values of *V* = 1 and *V* = 5. In Figure 3(b), at *V* = 1, when there is no quarantine (*β*_2_ = 0.0), the peak of *I*(*t*) ~ *O*(10^7^), and when the quarantine rate *β*_2_ ≥ 0.5, about 50 infected population is present in our simulation results. In Figure 3(c), at *V* = 5, when quarantine rate *β*_2_ = 0.0, the peak of *I*(*t*) ~ 500, and if *β*_2_ = 0.5, there are approximately 10 infected population in our simulation result. Thus, in Figure 3(b) and (c), the peak values of *I*(*t*) decrease as the quarantine rate *β*_2_ increases. For both system size values, if the quarantine process of the exposed population *E* is carried out as early as in 2 days, the Covid-19 transmission in Delhi is greatly controlled. Again, our stochastic simulation results highlight the importance of quick implementation of quarantine and social distancing to control the pandemic in a densely populated, small state like Delhi. The simulation results based on the available data indicate that the Covid-19 spreading in Delhi is not yet under control and that the imposition of strict intervention policies such as proper quarantine and social distancing must continue in the state.

*Scenario 4 (Kerala):* Our stochastic simulation results of Kerala (Fig. 3(d)–(f)) are quite impressive concerning the Covid-19 disease dynamics in the state. From Figure 3(d), the dynamics of *I*(*t*) for different values of the system size *V* show that the Covid-19-infected population *I*(*t*) decreases with an increase in *V*. When *V* = 1 (or *D* = *N*), the peak of *I*(*t*) ~ *O*(10^6)^ and when *V* ≈ 5 (or *D* ≈ *N*/5), the peak of *I*(*t*) ~ *O*(10^2^). We observed an overall decline in *I*(*t*) for all *V* ≥ 1 which indicates a proper intervention of the Covid-19 pandemic in Kerala. We thus found that decreasing the number density *D* of the population (maybe due to policies such as social distancing as mentioned earlier) at early times of the pandemic could be a good strategy for effectively controlling the pandemic. Again, in Figure 3(e) and 3(f), we studied the impact of quarantine (*β*_2_) on the time evolution of *I*(*t*) for two system size values *V* = 1 (or *D* = *N*) and *V* = 5 (or *D* = *N*/5). In Figure 3(e), at *V* = 1, when there is no quarantine (*β*_2_ = 0.0), the peak of *I*(*t*) ~ *O*(10^6^) and if the quarantine rate *β*_2_ = 0.5, then *I*(*t*) ~ 100 in our simulation result. Again, in Figure 3(f), at *V* = 5, when quarantine rate *β*_2_ = 0.0, the peak of *I*(*t*) ~ 250, and if *β*_2_ ≥ 0.5, then there are about 50 infected population in our simulation result. Thus, in Fig. 3(e) and (f), the peak values of the Covid-19-infected population *I*(*t*), at both values of *V*, decrease as the quarantine rate *β*_2_ increases. In Figure 3(e), if the exposed *E* population is quarantined in about 10 days, then the *I*(*t*) curve flattens. Again, in Figure 3(e), if the quarantine process is carried out in 2 days, then the *I*(*t*) curve shows a decreasing trend. Further, in Figure 3(f), the Covid-19 spreading in Kerala is greatly controlled if the exposed population *E* is quarantined in 2 days. These results again point out the importance of quarantine and social distancing as early as possible during the Covid-19 outbreak. Hence, the Covid-19 transmission in Kerala is quite controlled as compared to Uttar Pradesh and Delhi as per data.

*Scenario 5 (Maharashtra):* Maharashtra is the third-largest Indian state. Figure 4(a) shows the dynamics of the Covid-19-infected population *I*(*t*) for different values of the system size *V*, simulated using SSA. When *V* = 1 (or *D* = *N*), the peak value of *I*(*t*) ~ *O*(10^7^), indicating a Malthusian character in *I*(*t*). However, when *V* ≈ 5 (or *D* ≈ *N*/5), the *I*(*t*) curve flattens around *I*(*t*) ~ *O*(10^3^). Thus, for *V* ≥ 5 (or *D* ≤ *N*/5), the spread of Covid-19 is quite controlled. We observed the effect of increasing *V* or decreasing the number density *D* of the population in controlling the infected population *I*(*t*). In Figure 4(b) and (c), the time evolution of *I*(*t*) is again studied for different quarantine rates at two values of *V* = 1 (or *D* = *N*) and *V* = 5 (or *D* = *N*/5). In Figure 4(b), at *V* = 1, when there is no quarantine (*β*_2_ = 0.0), the peak of *I*(*t*) ~ *O*(10^7^), and if the quarantine rate *β*_2_ ≥ 0.5, then *I*(*t*) ~ 50 infected population in our simulation result. In Figure 4(c), at *V* = 5, when quarantine rate *β*_2_ = 0.0, the peak of *I*(*t*) ~ 450, and if *β*_2_ ≥ 0.5, roughly 50 infected population is present in our simulation result. The simulation results show that the dynamics of *I*(*t*) follow the Malthusian law for smaller values of *β*_2_ and that the *I*(*t*) curves start flattening for significantly larger values of *β*_2_, indicating a controlled behaviour of the pandemic. As discussed above, if the quarantine process of the exposed population *E* is carried out as early as possible during the Covid-19 outbreak, then the number of infected population *I*(*t*) can be significantly reduced. Since the *I*(*t*) curves for Maharashtra follow a Malthusian character, the Covid-19 pandemic situation in such a large, densely populated state is alarming. Stringent strategies such as social distancing, proper quarantining, etc., must continue for a longer time for proper intervention.

*Scenario 6 (West Bengal):* In the case of SSA simulation results of West Bengal-based data (Fig. 4(d)), when *V* = 1 (or *D* = *N*), the peak of *I*(*t*) ~ *O*(10^8^). However, for *V* ≈ 5 (or *D* ≈ *N*/5), the *I*(*t*) curve flattens about *I*(*t*) ~ *O*(10^2^), and for *V* > 5 (or *D* < *N*/5), the *I*(*t*) curves decline. Further, in Figure 4(e) and (f), we studied the effect of quarantine on *I*(*t*) evolution for two system size values *V* = 1 (or *D* = *N*) and *V* = 5 (or *D* = *N*/5). In Figure 4(e), at *V* = 1, when there is no quarantine (*β*_2_ = 0.0), the peak of *I*(*t*) ~ *O*(10^7^), and if the quarantine rate *β*_2_ ≥ 0.5, then *I*(*t*) ~ 50 infected population in our simulation result. Again, in Figure 4(f), at *V* = 5, when the quarantine rate, *β*_2_ = 0.0, the peak of *I*(*t*) ~ 150, and if *β*_2_ ≥ 0.1, there are about 10 infected population in our simulation result. From all these results, we observed that if proper quarantine of the exposed population *E* is not strictly imposed (smaller values of *β*_2_ < 0.1), then the increase in infected population *I*(*t*) is quite large, and flattening of the curves takes a long time (100–200 days). However, if proper quarantine of the *E* population is carried out (larger values of *β*_2_ ≥ 0.1), then the dynamics of *I*(*t*) shows decreasing trends, implying the Covid-19 pandemic is under control. The data-based simulation indicates that the scenario of West Bengal is still alarming if proper intervention is not taken.

**Fig. 5.**
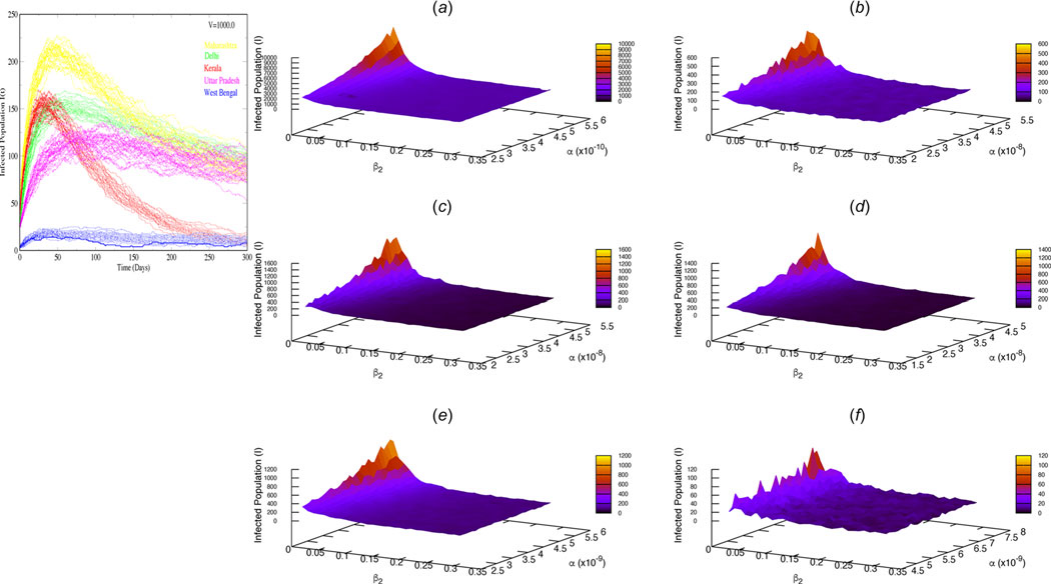
Left panel: Simulation results of *I*(*t*) *vs.* time *t* (in days) using Stochastic Simulation Algorithm for five Indian states, namely Maharashtra, Delhi, Kerala, Uttar Pradesh and West Bengal at a fixed system size *V* = 1000. Right panel: Heat Maps for (a) India, (b) Uttar Pradesh, (c) Delhi, (d) Kerala, (e) Maharashtra and (f) West Bengal to study the variation of the infected population *I* w.r.t the transmission rate *α* and quarantine rate *β*_2_. Stochastic fluctuations in *I* are clearly visible.

*Disease spreading pattern:* Using SSA, we again simulated the dynamics of *I*(*t*) for all the five Indian states as discussed above. We performed 30 realisations for each state. The infected population *I*(*t*) trajectories show peaks around 50 days and start declining (the left panel of Figure 5). However, we observed that except for Kerala and West Bengal, the decreasing trends in *I*(*t*) for Uttar Pradesh, Delhi and Maharashtra do not approach *I* = 0 over 300 days. This implies that social distancing and quarantine are not sufficient to bring an end to the Covid-19 pandemic. We then studied how Covid-19 spreads in the parameter space (*α*, *β*_2_, *I*) for India as well as the mentioned five states, as shown in the heat maps of Figure 5(a)–(f) (the right panel of Fig. 5). These heat maps are generated using GNUPLOT. From these heat maps, we observed that the Covid-19-infected population, *I* is relatively large for higher values of *α* and smaller values of *β*_2_. As the value of *β*_2_ increases, the *I* population drops. Hence, in order to control the Covid-19 pandemic in India and the five states, the parameters *α* and *β*_2_ need to be optimised. Moreover, we observed stochastic fluctuations in these heat maps. These stochastic fluctuations are known as demographic stochasticity which arises because of random births and deaths of individuals in populations. Demographic stochasticity plays an important role during the early stage as well as the ending stage of a disease outbreak when there are less infective individuals.

## Conclusion

Most of the epidemic models concerning the Covid-19 pandemic are studied using deterministic approaches which fail to capture the fluctuations/noise present in the system dynamics. We have studied the SEQIR model in the context of Covid-19 disease dynamics in India and five seriously affected states using stochastic methods. At an early stage of the Covid-19 outbreak in India, even though the basic reproduction number *R*_0_ values are calculated to be less than unity from the deterministic analysis, we can observe with stochastic modelling that the Covid-19 spreading in India and its five states can be disastrous if proper interventions are not put into effect as early as possible. Our numerical simulation results clearly show that policies like social distancing and quarantine have important roles in controlling the pandemic. We propose strict impositions of these two policies to effectively intervene in the Covid-19 disease transmission in the country as well as in the five states. An important consequence of employing a stochastic method is the appearance of demographic fluctuations in the simulation results to affect the disease dynamics and to even intervene in the spread of the disease. This demographic stochasticity which is quite important in regulating any system dynamics is generally neglected in its deterministic counterpart. Hence, our stochastic simulation method could capture the demographic stochasticity which is non-negligible. We would like to mention that we do not intend to give quantitative predictions here. One limitation of the model under consideration is that, by construction, *S* and *E* populations make transitions to the *Q* compartment where they are assumed to interact homogeneously. This may give rise to more infected populations and thus, we fail to see the trends of *I*(*t*) converging near zero over 300 days in our simulation results. Our study also points out that only policies such as social distancing and quarantine of the exposed population are not sufficient enough to end the Covid-19 pandemic in India and its states. Other stringent policies like complete lockdown as well as increased testing of susceptible populations must be considered and also incorporated systematically in mathematical models.

## Data Availability

Indian Covid-19 data are publicly available. Our simulation data are already included in this manuscript itself.
